# Hearing Function, Degeneration, and Disease: Spotlight on the Stria Vascularis

**DOI:** 10.3389/fcell.2022.841708

**Published:** 2022-03-04

**Authors:** Matsya R Thulasiram, Jacqueline M Ogier, Alain Dabdoub

**Affiliations:** ^1^ Department of Laboratory Medicine and Pathobiology, University of Toronto, Toronto, ON, Canada; ^2^ Biological Sciences, Sunnybrook Research Institute, Sunnybrook Health Sciences Centre, Toronto, ON, Canada; ^3^ Department of Otolaryngology–Head and Neck Surgery, University of Toronto, Toronto, ON, Canada

**Keywords:** single-cell sequencing, regeneration, cochlear battery, blood-labyrinth barrier, cisplatin, virus, clinical trial

## Abstract

The stria vascularis (SV) is a highly vascularized tissue lining the lateral wall of the cochlea. The SV maintains cochlear fluid homeostasis, generating the endocochlear potential that is required for sound transduction. In addition, the SV acts as an important blood-labyrinth barrier, tightly regulating the passage of molecules from the blood into the cochlea. A healthy SV is therefore vital for hearing function. Degeneration of the SV is a leading cause of age-related hearing loss, and has been associated with several hearing disorders, including Norrie disease, Meniere’s disease, Alport syndrome, Waardenburg syndrome, and Cytomegalovirus-induced hearing loss. Despite the SV’s important role in hearing, there is still much that remains to be discovered, including cell-specific function within the SV, mechanisms of SV degeneration, and potential protective or regenerative therapies. In this review, we discuss recent discoveries elucidating the molecular regulatory networks of SV function, mechanisms underlying degeneration of the SV, and otoprotective strategies for preventing drug-induced SV damage. We also highlight recent clinical developments for treating SV-related hearing loss and discuss future research trajectories in the field.

## 1 Introduction

**FIGURE 1:**
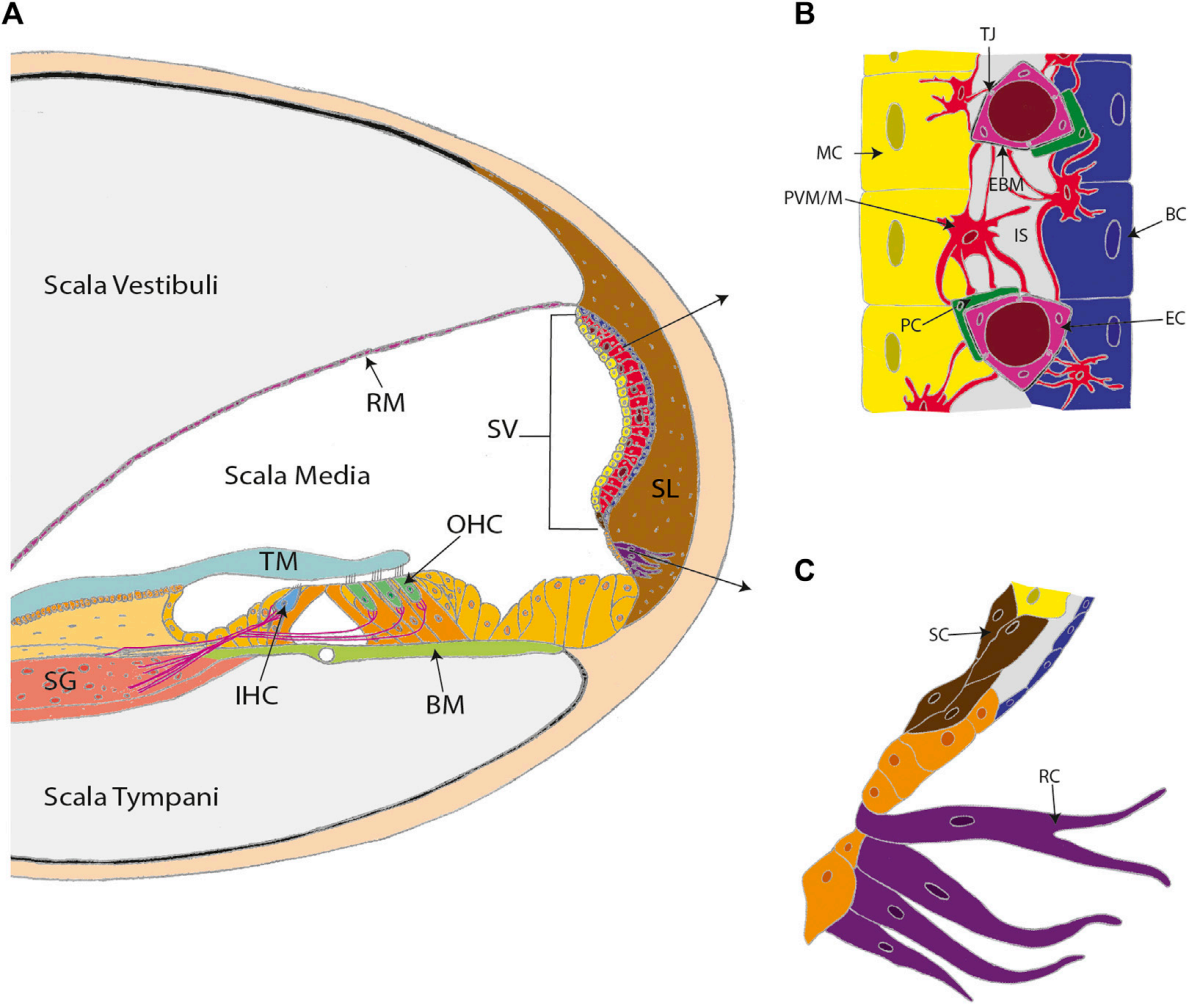
The stria vascularis (SV) is a highly specialized and vascularized tissue lining the lateral wall of the cochlea. **(A)** A cross-section of the cochlea. **(B)** The three conventional cell layers of the SV include the marginal cell layer (MC), the intermediate cell layer, and the basal cell layer (BC). The marginal cell layer is exposed to the endolymph and the basal cell layer interacts with spiral ligament fibrocytes. The intermediate cell layer is composed of perivascular-resident macrophage-like melanocytes (PVM/M), pericytes (PC), and endothelial cells (EC). The cell layers are tightly interlocked by infoldings and projections between the basolateral membranes of marginal cells and the PVM/Ms, and the PVM/Ms and the basal cells. **(C)** Other cell types in the lateral wall that contribute to SV function include spindle cells (SC) and root cells (RC). Other labels: Reisner’s membrane (RM); tectorial membrane (TM); spiral ganglion (SG); inner hair cell (IHC); outer hair cells (OHC); basilar membrane (BM); spiral ligament (SL); tight junctions (TJ); endothelial basement membrane (EBM) and intrastrial space (IS). **(C)** adapted from Gu et al. (2020).

**FIGURE 2:**
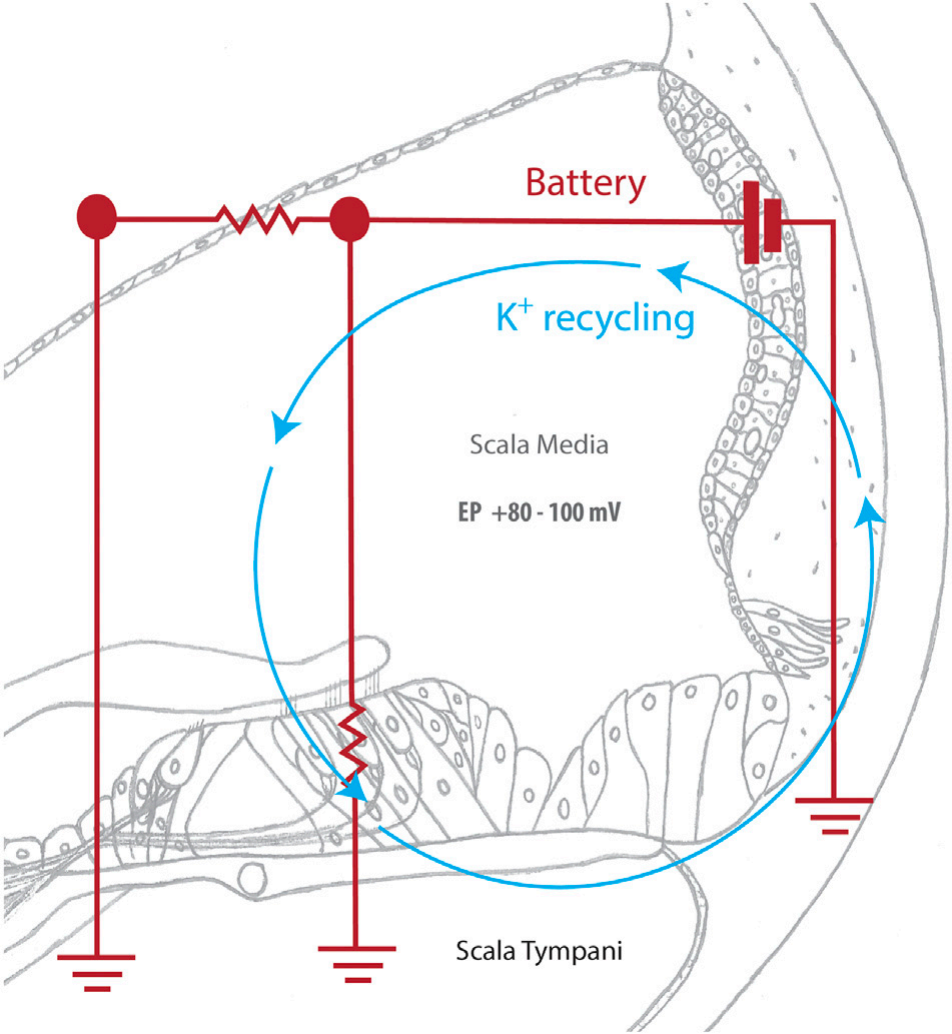
The cochlear battery facilitates sound detection. Sound waves deflect hair cell stereocilia, mechanically opening K+ channels and allowing potassium ions (K+) from the scala media to enter the hair cell. The subsequent depolarization of the hair cell activates voltage-gated calcium channels, which triggers a calcium influx that causes neurotransmitter to be released at the base of the hair cell. Neurotransmitter then diffuses into the nerve terminal and causes an action potential to be created in the spiral ganglion. This signal is then transmitted to the brain for auditory processing. The SV actively returns potassium ions that were used during this process to the scala media, maintaining the endocochlear potential and allowing for continuous sound detection.

The stria vascularis (SV) is a highly vascularized tissue located in the lateral wall of the cochlea that contributes to cochlear homeostasis in two ways (Figure 1). First, ion transport proteins in the SV perform active potassium ion (K^+^) recycling between the endolymph of the scala media and the perilymph of the scala tympani. This maintains the endolymph at a high K^+^ (∼157 mM), low sodium (Na^+^; ∼1.3 mM) state, that contrasts with the low K^+^ (∼4.2–6.0 mM), high Na^+^ (∼141–148 mM) state of perilymph (Liu et al., 2017; reviewed in Wangemann 2002, Wangemann 2006). This contrasting ionic composition creates an electric potential difference of +80–100 mV between the cochlear fluids, known as the endocochlear potential (Nin et al., 2016). For this reason, the SV is often referred to as the cochlear battery (Figure 2).

The SV directly contributes to hearing function by maintaining the endocochlear potential, which is required for the process of hair cell transduction to occur. Hair cell transduction is the critical point of sensing sound, where the mechanical sound wave is converted by cochlear hair cells into an electrochemical signal for auditory perception. This detection process begins when sound waves stimulate mechanically gated K^+^ channels in the hair cell stereocilia. Once open, these channels allow for the influx of K^+^ and the depolarization of the hair cell (Zhang Q et al., 2021). Subsequently, voltage-dependent calcium ion (Ca^2+^) channels are activated, allowing for the influx of Ca^2+^ and release of neurotransmitters, creating a signal that is sent to and processed by the brain (Gill and Salt 1997; Wangemann 2006). Once the hair cell is depolarized, K^+^ exits the hair cell and is taken up by the fibrocytes of the spiral ligament (reviewed in Zdebik et al., 2009). Gap and tight junction proteins, such as connexin and claudin11, then transport K^+^ into the SV, where ion transport channels, including the K^+^ inwardly-rectifying channel Kir4.1, traffic K^+^ through the SV layers, and back into the endolymph (Wangemann 2006). This active K^+^ recycling ensures that the endocochlear potential is maintained for sound detection to occur (Figure 2).

In addition, the SV contains the cochlear blood-labyrinth barrier (BLB), that tightly controls the transfer of material from the SV capillary network into the endolymph (Figure 1B). The BLB is often compared to the blood-brain barrier (BBB), with both vascular networks creating a physical barrier that limits paracellular diffusion and prevents infiltration of pathogens into the cochlea or brain respectively (Inamura and Salt 1992). Paracellular diffusion across the BBB is limited to small lipophilic or gaseous molecules (molecular weight <400–500 Da), whereas BLB permeability appears to extend beyond 500 Da (Dulon et al., 1986). Notably, hydrophilic aminoglycosides are unable to cross the BBB, whereas aminoglycosides such as tobramycin (467.515 Da), amikacin (585.6 Da), and gentamicin (477.596 Da) are readily transported via the BLB into the cochlea (Nau et al., 2010). Other known differences between the BBB and BLB include the type of supporting cells present (astrocytes vs. perivascular resident macrophage-like melanocytes in the BBB and BLB respectively) and basement membrane composition (reviewed in Nyberg et al., 2019). Nevertheless, further research is required to define the specific cell types and diffusion properties of the BLB. The functional complexity of the SV, combined with its difficult to access location within the temporal bone, has hindered SV research. However, advances in sequencing technology and the investigation of SV-associated disease mechanisms are improving our understanding of the SV. This review summarizes notable discoveries regarding SV function and novel opportunities that have been identified for developing SV-associated hearing loss treatments.

## 2 Physiology of the Stria Vascularis

The SV has three cellular layers, with each layer performing a specific function (Figure 1B). The marginal cell layer is exposed to the endolymph, transporting K^+^ from the SV into the scala media (Wangemann 2002). The intermediate cell layer includes the BLB and abundantly expresses the Kir4.1 ion channels that facilitate K^+^ transport. The basal cell layer associates with the spiral ligament fibrocytes via junction proteins to control ion flow into the SV and prevent ion leakage between the cochlear chambers (Liu et al., 2017). During development, the layers of the SV originate from different cell lineages. The marginal cells arise from the otic epithelium, whereas the specialized intermediate cells known as perivascular-resident macrophage-like melanocytes (PVM/Ms) are derived from migratory neural crest cells (Kiernan et al., 2002). Basal cells are formed from the otic mesenchyme after the marginal and intermediate cell layers are established (Kiernan et al., 2002; Locher et al., 2015).

While knowledge of the SV layers remains limited, recent RNA sequencing and bioinformatic analysis have been used to further elucidate cell-specific gene expression in the SV. For instance, single-cell RNA sequencing of the adult CBA/J mouse SV identified previously unknown gene expression in the SV (Korrapati et al., 2019). Newly identified genes include the lipid transporter gene, ATP binding cassette G1 (*Abcg1*), and the Notch signaling pathway effector gene, Hes-related family BHLH transcription factor with YRPW motif (*Heyl*), expressed in marginal cells; the K^+^ inwardly-rectifying channel J13 (*Kcnj13*) and a VEGF receptor gene, neuropilin 2 (*Nrp2*), expressed in intermediate cells; and the SRY-Box transcription factor 8 (*Sox8*), and the steroid/thyroid hormone receptor gene, nuclear receptor 2F2 (*Nr2f2*), expressed in basal cells. Further evaluation of these genes will likely provide important insight into cell-specific roles within the SV. In addition, Korrapati et al. used the Pharos database (https://pharos.nih.gov/) to demonstrate that 26 of the newly identified SV genes can be targeted by 93 FDA approved drugs, opening immediate avenues for SV-specific therapeutics.

Beyond the three conventional layers of the SV, single-nucleus RNA sequencing has also been used to identify distinct transcriptional profiles for rare spindle and root cells (Figure 1C). Spindle cells express annexin 1 (*Anxa1*) and dipeptidyl peptidase like 10 (*Dpp10*), which are target genes for the bric-a-brac (BTB) domain and cap‘n’collar (CNC) homolog 2 (*Bach2*) transcription factor—a potent immune regulator (Gu et al., 2020). Therefore, spindle cells may contribute to immune regulation in the SV. In contrast, the expression of ion channel genes, such as the chloride/bicarbonate exchanger, solute carrier 26A4 (*Slc26A4*), and the K^+^ inwardly-rectifying channel J10 (*Kcnj10*) in root cells suggests a role in maintaining the endocochlear potential (Gu et al., 2020). Notably, this finding supports previous electrophysiological observations made by Jagger et al. demonstrating that root cells display weak, rectifying whole-cell currents indicative of K^+^ channel activity (Jagger et al., 2010). Furthermore, immunohistochemistry indicates colocalization of Kir4.1 (the protein encoded by *Kcnj10*) within root cells, providing further evidence that root cells contribute to K^+^ recycling in the cochlea (Jagger et al., 2010). Significant research efforts are now required to correlate transcriptional findings with cell-specific function *in vivo*. However, these findings highlight the advantage of sequencing technology for identifying regulatory networks and SV cell-specific markers. Indeed, the characterization of previously unknown gene expression patterns in the SV has already provided novel targets for SV therapies and will likely increase our understanding of how complex biological functions are performed within the SV.

### 2.1 Characterizing the Stria Vascularis Blood-Labyrinth-Barrier

As discussed above, the SV provides a physical protective barrier that regulates which molecules enter the cochlea. Currently, the BLB is poorly understood, which has hindered the development and delivery of therapeutics to the cochlea. However, research focusing on pericytes and macrophage-like-melanocytes in the SV has provided further information regarding the structure and function of the BLB.

#### 2.1.1 The Role of Pericytes in the Blood-Labyrinth Barrier

Pericytes are specialized cells present along the abluminal surface of capillaries throughout the body. Within the cochlea, pericytes contribute to angiogenesis (the formation of new blood vessels), vascular integrity, and blood flow control (Reviewed in Caporarello et al., 2019). Pericyte depletion (in a Cre-mediated diphtheria toxin inducible mouse model) causes reduced vascular density, variable vessel size, vascular leakage, and hearing loss (Zhang J et al., 2021). Notably, RTqPCR and immunohistochemical analysis indicated that vascular endothelial growth factor isoform A165 (VEGFA165) is particularly important for pericyte function. Subsequently, Zhang et al. used an adeno-associated viral vector to deliver *Vegfa165* to pericyte-depleted mice, which promoted vascular growth and pericyte proliferation, ultimately restoring the endocochlear potential and hearing. These findings are consistent with previous observations made by Wang et al. who also demonstrated that VEGFA165 stimulates new vessel formation in adult C57BL/6 mouse SV explant cultures (Wang et al., 2019). Combined, these studies provide evidence that pericytes have a critical role for maintaining SV integrity, and that VEGFA165 might be a therapeutic target for preventing hearing loss associated with SV degradation. Other potential targets include zona occludens-1 (ZO-1) and VE-cadherin, which are tight junction proteins expressed by SV pericytes and associated with BLB endothelial cell integrity (Neng et al., 2013).

#### 2.1.2 Perivascular Resident Macrophage-Like Melanocytes Have a Regulatory and Protective Role in the Stria Vascularis

PVM/Ms are a hybridized cell type in the lateral wall, expressing both macrophage and melanocyte markers, such as EGF-like module-containing mucin-like hormone receptor-like 1 (F4/80), CD68 molecule (CD68), integrin alpha M (CD11b), macrophage/monocyte antibody (MOMA2), glutathione S-transferase alpha 4 (GSTα4), glutathione S-transferase (GST), and Kir4.1 (Shi 2010; Zhang et al., 2012). PVM/M’s regulate tight junctions, interact with endothelial cells of the SV capillary network, and contribute to the maintenance of cochlear homeostasis (Zhang et al., 2012). Interestingly, immunofluorescent labelling of PVM/Ms using ionized calcium binding adaptor molecule 1 (Iba1) identified that PVM/M morphology differs according to age in cochlear cross-sections from human donors (Noble et al., 2019). Specifically, PVM/Ms have a higher number of processes and lower nuclear cytoplasmic volume in samples from younger individuals (age 20–65) when compared to samples from older individuals (age 68–89+). Such structural differences may have important functional outcomes, particularly regarding the cochlear immune response, which might contribute to age-related hearing loss. In addition, Noble et al. observed that PVM/M morphology differs between the lateral wall and the auditory nerve regions. PVM/Ms in the SV have multiple thin projections of processes, whereas PVM/Ms in the auditory nerve appear to be bipolar and filopodia-like (Noble et al., 2019). This may indicate that lateral wall PVM/Ms have a surveillance function, whereas PVM/Ms of the auditory nerve are more motile and active in an injury response.

Overall, the macrophage activity of PVM/Ms is well-accepted. However, unanswered questions remain regarding PVM/M melanocyte activity in the SV. PVM/Ms are melanin-producing cells and interesting connections have been made regarding SV melanin content and hearing protection. Melanin is thought to preserve the endocochlear potential through melanin-Ca^2+^ interactions (Bush and Simon 2007; Gill and Salt 1997; Liu and Simon 2005). As mentioned previously, neurotransmission is dependent on Ca^2+^ influx into the hair cell following depolarization. Therefore, Ca^2+^ homeostasis is crucial for hearing transduction, and it is hypothesized that melanin acts as a Ca^2+^ chelator to modulate endolymphatic Ca^2+^ concentrations. Notably, a comparison of hearing ability (based on ABR measurements) in transgenic NMRI mice that were either pigmented and expressing melanin (YRT2 mice), non-pigmented but expressing the melanin precursor L-DOPA (TyrTH mice), or albino non-transgenic NMRI littermates (NMRI), demonstrated that aging YRT2 and TyrTH mice exhibit significantly less hearing loss after noise exposure than NMRI littermates (Murillo-Cuesta et al., 2010). Interestingly, hearing thresholds between YRT2 mice and TyrTH mice did not significantly differ, suggesting that the presence of melanin precursor is sufficient to protect hearing. Furthermore, YRT2 and TyrTH mice had better hearing recovery following noise insult when compared to non-transgenic littermates. Similarly, pigmentation appears to have a protective role for hearing in humans. Using the Fitzpatrick skin colour scale and audiometry, Lin et al. showed that individuals with darker skin have better hearing than those with lighter skin (Lin et al., 2012). Skin pigmentation appears to correlate with melanin content in the SV, and Andresen et al. demonstrated that SV melanin is indeed higher in African Americans when compared to Caucasians (Andresen et al., 2021).

Overall, melanocytes appear to have a particularly important role for supporting hearing function. However, the mechanism of protection is not clear. Melanocytes could be protecting the ear by providing immune surveillance in the SV, regulating cochlear Ca^2+^ levels, controlling vascular permeability, or any combination thereof. Furthermore, melanocyte-secreted melanin is a free radical scavenger that can prevent damaging redox stress in the ear (Sichel et al., 1991; Wu et al., 2001). Therefore, clarifying the role of melanin in the ear is worthwhile, as it may be possible to protect the cochlea in damaging situations using melanin supplementation. However, an important observation is that melanin does not protect against age-related SV degeneration. In fact, melanin content increases in the aging ear of mice and humans despite the prevalence of age-related hearing loss (Kobrina et al., 2020; Andresen et al., 2021).

## 3 Stria Vascularis Dysfunction and Hearing Loss

The SV protects the cochlea and preserves cochlear homeostasis, directly contributing to auditory detection. Therefore, dysfunction of the SV can have severe consequences for hearing. In this section, we discuss how SV dysfunction underlies age-related hearing loss, hearing loss induced by ototoxic medicines, several forms of heritable hearing loss (Table 1), and viral infection-mediated hearing loss.

**TABLE 1:** Summary of SV disorders and associated clinical trials.

Disorder	Prevalence	Identified Causative Genes	Phenotype	Intervention/Clinical trial identification	References
Norrie disease	400 cases worldwide	*Ndp*	Vascular degeneration	N.A.	Berger et al. (1996)
					Rehm et al. (2002)
					Sowden et al. (2020)
Meniere’s disease	200–500 in 100,000	*Esrrb*	SNHL	NCT00802529	Gu et al. (2021)
		*Atp1b2*	Tinnitus	NCT03664674	Gürkov et al. (2016)
		*Ednrb*			
		*Tmem176A*		NCT04677972	Kil et al. (2022)
		*Slc44a2*		NCT03325790	Lambert et al. (2016)
		*Col1a2*			Patel et al. (2016)
Alport syndrome	1 in 5,000	*Col4a3*	Basement membrane dysfunction	NCT04947813	Dufek et al. (2020)
		*Col4a4*			
		*Col4a5*			Gratton et al. (2005)
Waardenburg Syndrome	2–3 in 100,000	*Mitf*	Loss of pigmentation	N.A.	Chen L. et al. (2016)
		*Pax3*			Chen L. et al. (2020)
		*Sox10*			Chen T. et al. (2018)
		*Ednrb*			Flesher et al. (2020)
		*Edn3*			Saleem, (2019)
		*Snai2*			Song et al. (2016)

### 3.1 Age Related Degeneration of the Stria Vascularis

**FIGURE 3:**
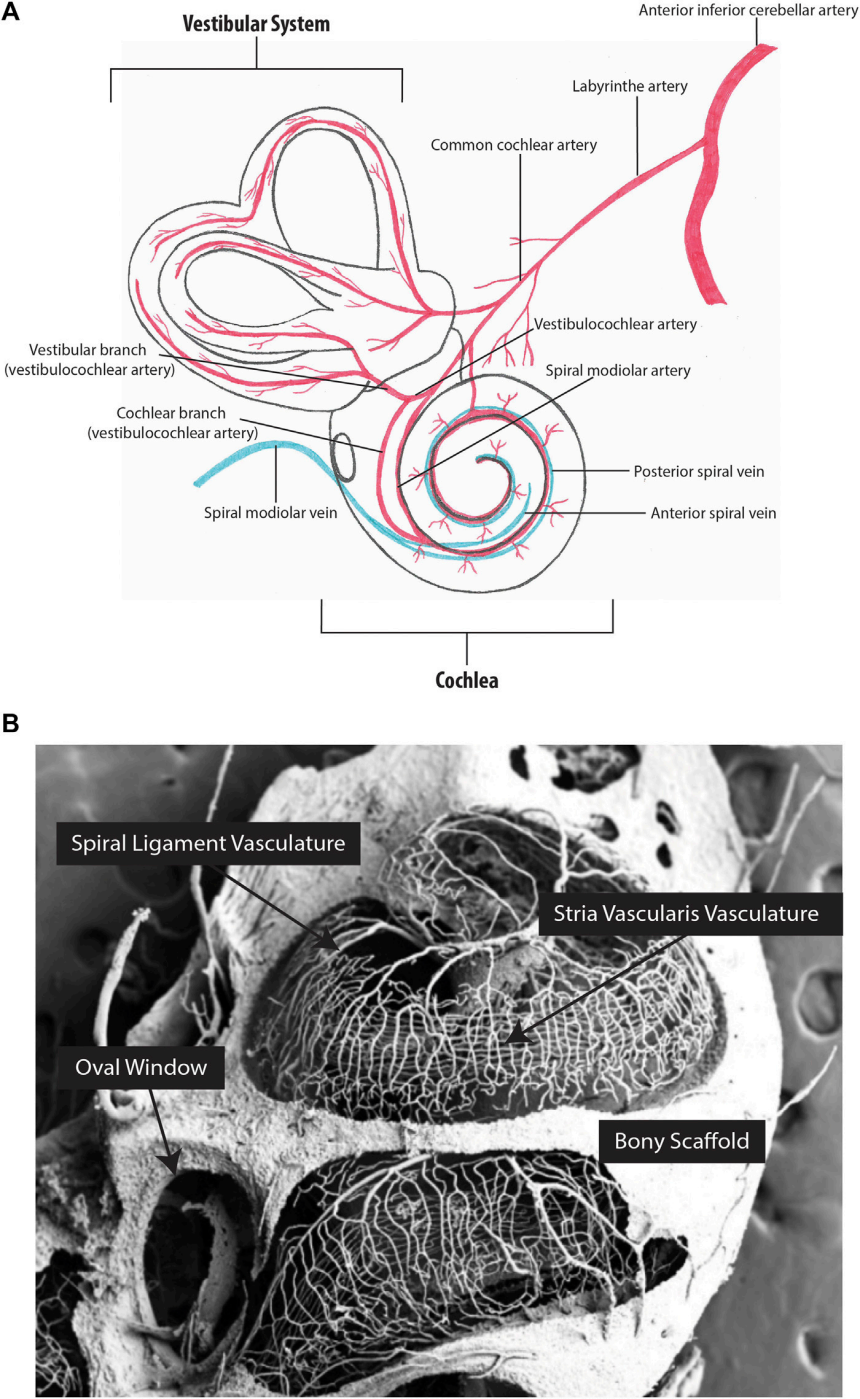
Inner ear vascular network. **(A)** Arterial blood supply and venous drainage of the cochlear and vestibular systems. **(B)** Partial corrosion casts of the mouse cochlea. The capillaries of the stria vascularis run parallel along the length of the cochlea. Figure 3B image provided by M. Carraro and R.V. Harrison.

Age-related hearing loss, known as presbycusis, affects over 25% of individuals above 60 years of age (World Health Organization 2021). Presbycusis has repeatedly been associated with the death of hair cells in the cochlea, and a widely recognized hypothesis suggests that degeneration of the SV, specifically the BLB and impaired SV blood flow, is the primary contributing factor (Figure 3A) (Schuknecht et al., 1974; Pauler et al., 1988; Gratton et al., 1996; McFadden et al., 1999; Gates and Mills 2005; Zhang et al., 2013; Carraro and Harrison 2016; Wang and Puel 2020). As previously discussed, tight regulation of ion channel function in the SV is an important aspect of endocochlear potential maintenance (reviewed in Peixoto Pinheiro et al., 2021). RTqPCR and immunohistochemical analysis of two essential players in the SV (Na+/K + ATPase and Kir4.1) in young (1.5–3-week-old) and aged (1.5–2.5-year-old) CBA/CaJ mice has demonstrated reduced ion channel expression in the SV of aged animals. Notably, reduced ion channel expression corresponded with dysregulation of the endocochlear potential and hearing loss (Ding et al., 2018; Liu T. et al., 2019). Similarly, in a guinea pig model of accelerated aging (induced by D-galactose), RTqPCR indicated reduced expression of the Ca^2+^-activated chloride channel, transmembrane protein 16A (*TMEM16A*) in the SV of aged guinea pigs (1-year-old) when compared to young guinea pigs (2-week-old). The reduced *TMEM16A* expression corresponded with increased hearing thresholds (Zhou et al., 2019). Combined, these studies demonstrate that the aging SV can no longer support the endocochlear potential due to the loss of important ion channels, and that this directly causes age-related hearing loss. Interestingly, this hypothesis was challenged by Wu et al. who analysed 120 human inner ears (obtained during autopsy) and concluded that hair cell death preceded SV atrophy (Wu et al., 2020). However, this study relied solely on histopathological evidence, which cannot detect critical changes in ion recycling or the endocochlear potential changes that likely cause the death of sensory hair cells. Moreover, a similar histopathological study by Kurata et al. identified increased vascular thickness, reduced vessel counts, and decreased total SV area in the SV of individuals with presbycusis when compared to age-matched controls, contradicting the results obtained by Wu et al. (Kurata et al., 2016). However, cochlear fluid evaluation is required to truly answer the question of whether SV degeneration drives age-related hearing loss. Unfortunately, sampling from the living cochlea is not possible, as it is invasive and risks inducing hearing loss. Therefore, mouse models have been utilized to further understand age-related degeneration of the SV. For example, Carraro and Harrison developed a partial corrosion-cast technique using C57BL/6 mice, to capture the full structural integrity of the cochlear vasculature using polymer perfusion (Figure 3B) (Carraro and Harrison 2016). Using this technique in conjunction with SEM, Carraro and Harrison showed that 6-month-old mice have significantly narrower vasculature within the basal turn of the SV when compared to 1-month-old mice, and that these changes correspond with increased high frequency ABR hearing thresholds. Immunohistochemical analysis of the BLB also identified a loss of pericytes and PVM/Ms in aged C57BL/6 mice which has been previously shown to cause vascular leakage (Neng et al., 2015). However, C57BL/6 mice carry the cadherin 23 mutation, which is a well-established cause of age-related hearing loss (Sotomayor et al., 2010). Accordingly, Kobrina et al. used CBA/CaJ mice (which have more robust hearing) to assess hearing and cochlear histopathology during the aging process (Kobrina et al., 2020). Aged mice demonstrated decreased behavioural sound detection, increased ABR hearing thresholds, decreased hair cell survival, reduced SV thickness, and increased SV melanin when compared to young mice. Taken together, findings from mice and humans indicate that age-related changes in the SV microvasculature impact circulation and perfusion within the cochlea, contributing to functional changes in hearing. However, further mechanistic investigations are required to confirm these observations. Using the data obtained from single cell sequencing, new conditional knockout mouse models can be created to study the role of specific genes in the SV. These will provide insights into the mechanisms by which the SV develops, degenerates with age, and possible means of prevention and regeneration.

### 3.2 Ototoxicity and the Stria Vascularis

Ototoxicity is the result of a substance damaging any part of the cochlea, vestibular system, or auditory nerve. While ototoxic medicines are generally avoided in the clinic, their use is often required as part of a life-saving medical intervention. Three ototoxic drug classes are particularly problematic. First are the aminoglycoside antibiotics, used to manage chronic or multi-drug resistant infection. Aminoglycoside ototoxicity typically damages hair cells directly after being trafficked through the BLB into the endolymph (Imamura and Adams 2003; Li and Steyger 2011; Wang and Steyger 2009). Secondly, loop diuretics, which are used to treat hypertension and edema, directly target the SV. Loop diuretics, such as ethacrynic acid, cause a transient reduction of the endocochlear potential due to disrupted SV blood flow and inhibited Na^+^/K^+^ ATPase and Na^+^/K^+^/Cl^−^ cotransporter (NKCC1) function (Ding et al., 2016). Lastly, the platinum-based chemotherapeutics, which are used to treat a range of metastatic and refractory tumours in adults and children, appear to affect both the SV and the hair cells through long-term accumulation within the cochlea (Breglio et al., 2017). Several ototoxic platinum-based chemotherapeutics are used, including carboplatin, oxaliplatin, and cisplatin (Gersten et al., 2020). However, of these chemotherapeutics, cisplatin is the most widely used and the most ototoxic (Watanabe et al., 2002; Thomas et al., 2006). Cisplatin causes significant damage to hair cells, spiral ganglion neurons, and the SV, resulting in permanent hearing loss in both pediatric and adult recipients (Bertolini et al., 2004; Frisina et al., 2016). Inductively coupled plasma mass spectrometry shows long-term retention of cisplatin in the SV (Breglio et al., 2017) and cisplatin treatment reduces the endocochlear potential (Tsukasaki et al., 2000; Breglio et al., 2017). Immunofluorescent microscopy indicates that cisplatin reduces SV expression of the tight junction protein, ZO-1, and gap junction proteins, Connexin26 and Connexin34 (Zhang N. et al., 2020). Furthermore, PVM/Ms are activated and damaged in response to cisplatin treatment. Overall, cisplatin broadly affects SV barrier integrity, impacting the endocochlear potential, and initiating an immune response that leads to long-term SV damage, resulting in hearing loss. Therefore, given the necessity of platinum-based therapeutics for life-saving cancer treatment, there is a significant need for otoprotective substances that can prevent SV damage.

#### 3.2.1 Protecting the Ear From Ototoxicity

The vast majority of proposed otoprotective compounds target redox stress specifically within the hair cell. However, antioxidants may also reduce redox stress within the SV, providing further protection for the cochlea. For example, Cai et al. investigated apoptotic events in the SV driven by cisplatin treatment and examined the protective effect of allicin, a natural lipophilic small molecule antioxidant (Cai et al., 2019). Mice were pre-treated with allicin twice, receiving 18.2 mg/kg intraperitoneally 1 day before, and then 2 hours before starting cisplatin treatment. Intraperitoneal cisplatin was then administered once daily for seven consecutive days at 3 mg/kg. Cisplatin treatment caused pro-apoptotic factors such as Poly [ADP-ribose] Polymerase 1 (PARP-1) and apoptosis inducing factor (AIF) to be expressed in the SV. However, PARP-1 and AIF expression was significantly reduced in allicin pre-treated mice. This indicates that allicin might be an otoprotective compound. However, functional hearing assessments were not performed. Furthermore, there is a risk that antioxidant use can reduce chemotherapeutic efficacy (Lawenda et al., 2008). Therefore, significantly more research is required to ascertain whether allicin treatment is a strategy for reducing cisplatin-associated ototoxicity. Alternatively, statin medications have shown otoprotective promise against platinum-based drug and aminoglycoside-induced ototoxicity (Brand et al., 2011), as well as noise-induced hearing loss (Park et al., 2012; Richter et al., 2018). Statin medications are cholesterol-lowering drugs that inhibit 3-hydroxy-3-methylglutaryl-CoA (HMG-CoA) reductase, the rate-limiting enzyme in cholesterol biosynthesis. Fernandez et al. predicted that statin otoprotection was due to reduced cisplatin uptake and retention within the SV (Fernandez et al., 2020). However, when testing this hypothesis, lovastatin treatment did not prevent high accumulation of cisplatin in the mouse SV (Fernandez et al., 2020). Therefore, the mechanism by which statins are otoprotective remains elusive. Nevertheless, a recent clinical observational study found that individuals taking atorvastatin with concurrent cisplatin therapy had significantly better hearing following cisplatin treatment compared to non-statin treated individuals (Fernandez et al., 2021; NCT03225157). Overall, 277 individuals with head and neck squamous cell carcinoma participated in the study, with 113 participants already receiving statin medication. Baseline audiometric thresholds for all participants were compared with follow up audiometry collected within 90 days of cisplatin treatment. High frequency threshold shifts were approximately 10 dB greater in participants receiving cisplatin alone when compared to those taking atorvastatin, and the otoprotective effect of atorvastatin was independent of the atorvastatin dose. Therefore, statins should be further evaluated as an otoprotective strategy, particularly given that statins are already FDA approved.

### 3.3 Heritable Forms of Hearing Loss Lead to Stria Vascularis Degeneration

#### 3.3.1 Norrie Disease

Norrie disease is a rare X-linked recessive disorder associated with mutations of the Norrie disease protein gene (*Ndp*, Xp 11.3; Berger et al., 1996). Only 400 cases of Norrie disease have been documented worldwide (Sowden et al., 2020), associated with more than 115 *Ndp* mutations (Yamada et al., 2001; Halpin et al., 2005; Allen et al., 2006; Parzefall et al., 2014; Andarva et al., 2018; Rodríguez-Muñoz et al., 2018). While each mutation causes a unique phenotype, severe vascular degeneration is a common feature of Norrie disease, contributing to sensory degradation and developmental deficits. Most individuals diagnosed with Norrie disease have congenital blindness, and while they may develop hearing loss at any time from childhood to late adulthood, the median age of onset is early adolescence (Halpin and Sims 2008). Norrie disease is often misdiagnosed due to the rarity of the condition and the complexity of symptoms. However, improved genetic testing is facilitating earlier diagnosis (Wu et al., 2017; Liu J. et al., 2019; Marakhonov et al., 2020). Nevertheless, even with an accurate diagnosis, it is not possible to predict when hearing loss may occur for individuals with Norrie disease.

The progression of Norrie disease-associated hearing loss has been investigated using *Ndp* knockout mice (Rehm et al., 2002). In this model, mice develop progressive hearing loss at 3 months of age, corresponding with enlargement of the SV microvasculature (Rehm et al., 2002). The three SV cell layers progressively degrade, leading to a collapse of the SV and total hearing loss by 15 months of age. The degeneration of SV blood vessels in *Ndp* knockout mice is also consistent with observations made in the vasculature of the brain (Wang et al., 2012) and the retina (Ye et al., 2009) of other *Ndp* loss-of-function mouse models. In addition, recent evidence from Hayashi et al. found that Ndp plays a role in cochlear hair cell survival and maturation (Hayashi et al., 2021). Functional assessment of hearing using distortion product otoacoustic emission (DPOAE) measurements revealed elevated thresholds indicative of hair cell dysfunction in Ndp knockout mice at 2 months of age. Further research is required to ascertain the molecular mechanisms driving degeneration in Norrie disease and how individual *Ndp* mutations might affect specific cell types. However, it appears that the canonical Wnt/ß-catenin signalling pathway is impacted by Norrie disease-associated mutations.

Canonical Wnt/ß-catenin signalling regulates cell-fate determination and angiogenesis during cochlear development (Geng et al., 2016). Wnt ligands bind to different frizzled (Fzd) receptors and low-density lipoprotein receptor-related protein (Lrp) co-receptors, allowing for the accumulation and subsequent translocation of intracellular ß-catenin to the nucleus to activate the T-cell factor/lymphoid enhancer factor (TCF/LEF) transcription factors (reviewed in Franco et al., 2009). Interestingly, Ndp is an atypical Wnt ligand which activates canonical Wnt/ß-catenin signalling by selectively binding to frizzled-4 (Fzd-4) at the same interaction site as typical Wnt ligands (Chang et al., 2015). In addition, Ndp-mediated Wnt/ß-catenin signalling requires the interaction of both low-density lipoprotein receptor-related protein 5/6 (Lrp5/6), and the transmembrane protein, tetraspanin 12 (Tspan12) to function (Lai et al., 2017). Currently, it is predicted that *Ndp* mutations result in tight junction loss and vascular barrier remodelling which may contribute to Norrie disease SV degradation due to impaired interactions between Ndp, Fzd-4, and Tspan12 (Xu et al., 2004; Lai et al., 2017). Recent observations made by Hayashi et al. indicate that *Ndp* loss-of-function also impacts hair cell development due to irregular ß-catenin signalling (Hayashi et al., 2021); however, the EP as a measure of SV function was not investigated. Characterization of this pathway in the inner ear may distinguish the importance of Ndp compared to Wnt in mediating Norrie disease-associated hearing loss and identify potential therapeutic targets.

#### 3.3.2 Meniere’s Disease

Meniere’s disease is a prominent hearing disorder affecting approximately 200–500 people per 100,000 (Gürkov et al., 2016). Meniere’s disease is characterized by chronic sensorineural hearing loss, vertigo, and tinnitus. Three-dimensional reconstruction and magnetic-resonance-imaging of the inner ear indicate that endolymphatic hydrops (EH), or the expansion of the endolymphatic space to occupy areas normally only containing perilymph, contribute to Meniere’s hearing loss (Morita et al., 2009; Naganawa et al., 2010; Naganawa and Nakashima, 2014). It is speculated that this is due to SV dysfunction, particularly an increased permeability of the BLB (Zhang W. et al., 2020).

Currently, there is no cure for Meniere’s disease and treatments to manage Meniere’s symptoms are limited. For severe cases of vertigo, the ototoxic aminoglycoside antibiotic, gentamicin, is injected intratympanically. This approach provides relief by destroying vestibular hair cells, which subsequently removes dysfunctional balance sensation. However, vestibular hair cell death impairs normal balance and intratympanic gentamicin can also damage cochlear hair cells, causing permanent hearing loss. Therefore, the corticosteroids methylprednisolone (NCT00802529) and dexamethasone (NCT03664674) have been tested in human clinical trials as potential gentamicin replacements (Lambert et al., 2016; Patel et al., 2016; Patel 2017). Encouragingly, methylprednisolone reduced the number of vertigo attacks experienced by individuals with Meniere’s disease and did not impact speech recognition to the same extent as gentamicin. Likewise, dexamethasone reduced the severity of vertigo, without causing hearing loss (Patel 2017). In addition, the FDA has recently approved a phase three clinical trial for *ebselen*, another potential Meniere’s therapeutic (NCT04677972; reviewed in Kil et al., 2022). *Ebselen* can be taken orally, avoiding painful intratympanic injections, and, while *ebselen* may not improve vertigo, it significantly reduces tinnitus and hearing loss in individuals with Meniere’s disease (as observed in phase two clinical trials: NCT03325790). While the mechanism of effect needs to be better defined, these therapeutic advances could significantly improve the outcomes experienced by individuals with Meniere’s disease, especially when compared to gentamicin treatment. In addition, a recent study by Gu et al. used published RNA-sequencing data in adult mice and compared it to published datasets regarding Meniere’s disease in humans to localize known Meniere’s disease genes within specific cells of the SV (Korrapati et al., 2019; Gu et al., 2021). Estrogen related receptor beta (*Esrrb*) and Na^+^/K^+^ transporting ATPase 1b2 (*Atp1b2*) expression was identified in marginal cells, endothelin receptor type B (*Ednrb*) and transmembrane protein 176A (*Tmem176A*) in intermediate cells, and solute carrier 44a2 (*Slc44a2*) and collagen type 11 a2 (*Col11a2*) in basal cells. Future research to determine the involvement of these genes in Meniere’s disease and SV function may improve Meniere’s disease models and identify novel therapies.

#### 3.3.3 Alport Syndrome

Alport syndrome is a rare inherited type IV collagen disorder, affecting approximately 1 in 5,000 people (Gratton et al., 2005). Alport syndrome symptoms include kidney failure and late-onset progressive sensorineural hearing loss, caused by the progressive thickening of the SV capillary basement membrane and the dysregulation of extracellular matrix proteins (Thomopoulos et al., 1997; Gratton et al., 2005; Cosgrove et al., 2008; Meehan et al., 2016). Mutations in collagen type 4 alpha 3, (COL4A3), collagen type 4 alpha 4 (COL4A4), and collagen type 4 alpha 5 (COL4A5) have been associated with Alport syndrome.

Notably, the COL4A4-knockout mouse has been used to demonstrate that SV function is impaired in the Alport syndrome model (Cosgrove et al., 1996). Specifically, SV capillary permeability is reduced in 8.5-week-old COL4A4 knockout mice when compared to age matched wild-type mice (demonstrated using intracardially injected Rhodamine dye and fluorescence spectrometry) (Dufek et al., 2020). In addition, RNA sequencing showed higher expression of inflammatory genes, such as nuclear factor kappa B inhibitor alpha (*NFKBIA*), in the SV of COL4A4 knockout mice when compared to wildtype controls. This would suggest that the host’s inflammatory response damages the SV in those with Alport syndrome (Dufek et al., 2020). It remains to be seen what is causing this inflammation in the Alport syndrome-affected SV. However, inflammation is certainly associated with increased vascular permeability, indicating that Alport syndrome-associated SV degeneration and subsequent BLB degradation causes Alport syndrome-associated hearing loss.

Very little data is available regarding SV function in humans with Alport syndrome. However, an observational clinical trial is evaluating Alport syndrome-associated symptoms in people with a history of renal hematuria, and next generation sequencing confirmed COL4A mutations over a period of 4 years (NCT04947813). This study will produce important human-based information, but more research is needed in both animal models and humans to identify SV pathology so that treatments may be developed.

#### 3.3.4 Waardenburg Syndrome

Waardenburg syndrome is a rare auditory-pigmentary disorder affecting 2-3 individuals per 100,000 (Saleem 2019). There are four types of Waardenburg syndrome, classified by symptom and primarily caused by six genetic mutations: melanocyte inducing transcription factor (*Mitf*), paired box 3 (*Pax3*), SRY-box transcription factor 10 (*Sox10*), Ednrb, endothelin 3 (*Edn3*), and snail family transcription factor 2 (*Snai2*) (reviewed in Song et al., 2016). Individuals with Waardenburg syndrome can experience a loss of pigmentation in their hair, eyes, skin, and melanocytes of the SV. As previously discussed, the protective role of melanocytes in the SV is not yet clear. Notably, research regarding Waardenburg-associated gene function has identified important regulatory factors that may be used to further elucidate the function of melanin-producing cells in the SV. For example, *Mitf* interacts with melanogenesis-associated enzymes, including tyrosinase and dopachrome tautomerase (Chen et al., 2018; Flesher et al., 2020). A *de novo* mutation in the *Mitf* promoter isoform, *Mitf-M*, causes pigmentation loss and hearing damage in mouse and porcine models, indicating that a loss of *Mitf-M* might hinder melanogenesis in the SV (Chen et al., 2016). Furthermore, RNA sequencing of the SV collected from *Mitf-M* animals also indicates that ion transport genes such as transmembrane receptor cation channel M1 (*Trpm1*), *Kcnj13*, and solute carrier 45A2 (*Slc45a2*) are downregulated (Chen et al., 2020). These results suggest that melanin-producing cells in the SV likely contribute to the ionic regulation of the cochlear fluid, however, ionic measurements are required to confirm this hypothesis. Furthermore, investigation of the candidate genes identified by RNA sequencing in Waardenburg syndrome animal models should be pursued to evaluate potential drug targets for protecting the melanocytes of the SV.

Interestingly, the MITF protein is a downstream regulatory factor of the Wnt/ß-catenin signaling pathway that interacts with the LEF-1 transcription factor (Takeda et al., 2000; Saito et al., 2002; Yasumoto et al., 2002). Therefore, combined with the observation that Wnt/ß-catenin signaling is impacted by Norrie disease associated mutations, it appears that the Wnt pathway has a prominent role for SV development and function.

### 3.4 Cytomegalovirus-Induced Hearing Loss

Congenital Cytomegalovirus (CMV) infection is one of the most common causes of pediatric sensorineural hearing loss. 35% of infants with symptomatic CMV and 7–10% of infants with asymptomatic CMV will develop hearing loss (Manicklal et al., 2013). The mechanism of CMV-induced hearing loss is not clear. However, studies in mice and humans indicate that CMV infection causes SV degeneration, which subsequently causes hearing loss. For example, when newborn BALB/c mice are inoculated with murine CMV, increased BLB permeability can be observed using the evans blue tracer assay as early as 3 weeks post infection. Corrosion casts have also been used to identify severe structural damage to the vessels of the SV 8 weeks post CMV infection (Carraro et al., 2017) and mature mice have increased ABR thresholds when compared to uninfected controls (Li et al., 2014; Carraro et al., 2017). Carraro et al. hypothesized that CMV induced SV structural damage is caused by viral accumulation in the cochelar bone marrow, that may then impact PVM/M differentiation (Bradford et al., 2015; Carraro et al., 2017). As discussed above, PVM/Ms are bone marrow-derived cells (Shi 2010) critical for the regulation of SV vascular permeability and immune surveillance. Therefore, PVM/M loss likely disrupts blood flow, ion homeostasis, and the endocochlear potential in the CMV infected ear. Conversely, Bradford et al. speculated that CMV-induced SV degeneration might be caused by inflammation and tested whether murine CMV induced an inner ear inflammatory response in intraperitoneally infected newborn BALB/c mice (Bradford et al., 2015). 11 days post-infection, Bradford et al. observed CD3 positive cells in the SV and the spiral ganglion neurons. CD3 (cluster of differentiation three) is a T-lymphocyte coreceptor involved in immunoregulation. Therefore, the presence of CD3^+^ cells is indicative of an inflammatory response occurring in the SV in response to CMV infection. Likewise, CD8^+^ T lymphocytes have been observed near the sites of viral inclusion in electively terminated human fetuses (21-weeks gestational age) with congenital CMV infection (Gabrielli et al., 2013). Notably, in these fetuses, CMV DNA was observed in the marginal cells of the SV and Reisner’s membrane, indicating that CMV can infect and damage the SV, and subsequently enter the cochlea. In addition, CMV-induced hearing loss has been associated with oxidative stress. Pecha et al. used a superoxide-sensitive fluorescent probe to examine the presence of reactive oxygen species (ROS) 7 days post-CMV-infection in neonatal BALB/c mice (Pecha et al., 2020). Increased ROS production occured in the CMV-infected spiral ganglia, osseous spiral lamina, and SV when compared to uninfected controls.

There is no cure for CMV-induced hearing loss. However, valganciclovir, an FDA approved, orally administered, anti-viral medication prevented CMV induced hearing loss in a phase three clinical trial (NCT00466817; Kimberlin et al., 2015). During the trial, infants (≤30 days old) with symptomatic congenital CMV received oral valganciclovir treatment, with short- and long-term treatments evaluated. For short-term administration, 49 participants received valganciclovir for 6 weeks, followed by a placebo for 18 weeks. For long-term administration, 47 participants received valganciclovir treatment for 6 months. Hearing was assessed using brainstem evoked response measurements at baseline, 6, 12, and 24 months. Participants in both treatment groups showed similar hearing outcomes at 6 months when compared to baseline. However, a greater number of participants in the long-term treatment cohort had improved hearing at 12- and 24-months than those receiving short-term treatment. Overall, improved hearing outcomes were seen in 78 and 86% of participants at 12- and 24-months respectively in the long-term treatment cohort, compared to 63 and 71% of participants in the short-term treatment cohort. Notably, valganciclovir decreased the participants’ viral load in both treatment groups. However, discontinuation of valganciclovir in the short-term treatment group resulted in a CMV resurgence. This likely accounts for the poorer hearing outcomes observed in the short-term treatment cohort and highlights the need for prolonged valganciclovir treatment. Similar positive outcomes were observed by Bilavsky et al. in infants born with CMV and hearing impairment that were subsequently treated with valganciclovir for 12 months. After 12 months of valganciclovir treatment, 65% of the impaired ears had improved, with 76% of the improved ears returning to normal hearing levels (Bilavsky et al., 2016). Therefore, valganciclovir should certainly be considered for routine CMV treatment. In addition, valganciclovir and other newly developed anti-viral drugs may prove beneficial for treating other infections that cause hearing loss. While this remains to be tested, given the prevalence of hearing loss-inducing viruses and their associated mortality rates (reviewed in Cohen et al., 2014) the development of anti-viral drugs is a lifesaving and life-changing discovery. Indeed, for the children in both the Kimberlin and Bilavsky studies, the prevention of CMV-induced hearing loss will significantly improve their quality of life.

## 4 Conclusion and Future Directions

The SV has a vital role for hearing. It regulates the strict ionic composition of cochlear fluids and produces the endocochlear potential required for sound transduction, while also protecting the cochlea by providing immune surveillance and maintaining the BLB. However, the BLB remains one of the most under studied aspects of the ear, despite being one of the most important targets for delivering inner ear therapeutics. Nevertheless, single-cell sequencing technologies, combined with human focused histology and functional hearing assessments have significantly improved our understanding of the SV. Novel SV cell types have been identified and known cell types are proving to have more dynamic roles than previously thought. Direct avenues for clinical intervention have been identified, such as gene networks that can be targeted by FDA approved therapeutics. Follow up studies using knockout models to evaluate the importance of genes identified by single nucleus RNA sequencing will also provide greater detail regarding cell-specific roles in the SV. Furthermore, evaluation of these SV cell-specific genes might also identify biomarkers for diagnosing or monitoring SV health. To date, only a few inner ear biomarkers have been evaluated, but none are specific to the SV (Mahshid et al., 2021).

While several questions remain regarding SV development and function, significant groundwork has set the scene for burgeoning discovery. It is likely that this will subsequently facilitate the development of research tools such as organoids or organ-on-a-chip technologies, that have proven invaluable for high throughput drug screening and disease modelling in other fields. Overall, critical knowledge has been gained particularly elucidating the mechanisms of age-related SV degeneration, ototoxic outcomes, and SV-associated hearing disorders, providing exciting opportunities for preventing hearing loss in the future.
